# The impact of polygenic cardiovascular risk on Alzheimer’s‐like brain changes

**DOI:** 10.1002/alz.090777

**Published:** 2025-01-09

**Authors:** Sarah‐Alexis Lenhoff, Frederick A Page, Joseph Nowell, Paul Edison

**Affiliations:** ^1^ Imperial College London, London, Greater London UK; ^2^ Imperial College London, London UK

## Abstract

**Background:**

A growing body of evidence demonstrates the relationship between cardiovascular risk and the risk of dementia development. Researchers have identified several genetic links between cardiovascular diseases and Alzheimer’s disease, including the apolipoprotein E and methylenetetrahydrofolate reductase (MTHFR) genes. Here we evaluated the impact of cardiovascular polygenic risk on pathological brain changes associated with Alzheimer’s disease, to elucidate the potential mechanism by which cardiovascular risk induces brain damage.

**Methods:**

n ≈ 30,000 participants were evaluated from the UK Biobank database. The standard cardiovascular polygenic risk score was acquired for all participants. Where data was available the following data was downloaded; hippocampal volume and total grey matter volumes, white matter hyperintensity volume, whole brain grey matter and temporal cerebral blood flow, and diffusion‐weighted imaging outcomes. Linear regressions were performed to evaluate the influence of cardiovascular polygenic risk on brain changes in various regions implicated in Alzheimer’s disease.

**Results:**

A higher cardiovascular polygenic risk demonstrated the strongest association with an increased deep white matter hyperintensity volume. Increased polygenic risk was additionally associated with a reduced total grey matter volume. In the parahippocampal part of the cingulum, higher polygenic risk was associated with increased mean diffusivity and reduced orientation dispersion. No association was shown with cerebral blood flow.

**Conclusion:**

Genetic cardiovascular risk was demonstrated to be associated with several Alzheimer’s‐like neurodegenerative changes. A strong association was shown with white matter hyperintensities and decreased total brain volume, indicating the vulnerability to cognitive impairment in those with higher cardiovascular risk. Screening cardiovascular genetic risk at mid‐life could provide a useful tool to implement therapeutic intervention, not only to reduce the risk of cardiovascular disease but to prevent the development of Alzheimer’s disease.

